# Improvement of PMMA Dental Matrix Performance by Addition of Titanium Dioxide Nanoparticles and Clay Nanotubes

**DOI:** 10.3390/nano11082027

**Published:** 2021-08-09

**Authors:** Mariafrancesca Cascione, Valeria De Matteis, Paolo Pellegrino, Giovanni Albanese, Maria Luisa De Giorgi, Fabio Paladini, Massimo Corsalini, Rosaria Rinaldi

**Affiliations:** 1 Department of Mathematics and Physics “Ennio De Giorgi”, University of Salento, 73100 Lecce, Italy; paolopellegrino@unisalento.it (P.P.); marialuisa.degiorgi@unisalento.it (M.L.D.G.); fabio.paladini@le.infn.it (F.P.); ross.rinaldi@unisalento.it (R.R.); 2 U.O.C. of Plastic Surgery and Burns Center, Department of Oral Hygiene Clinic, Hospital “A. Perrino”, 72100 Brindisi, Italy; giannialbanese69@libero.it; 3 Dental School, Interdisciplinary Department of Medicine, University of Bari “Aldo Moro”, 70124 Bari, Italy; massimo.corsalini@uniba.it

**Keywords:** TiO_2_NPs, HNTs, poly(methyl methacrylate), oral prostheses, mechanical analysis, wettability, *Candida albicans*

## Abstract

Over the last decades, several materials have been proposed for the fabrication of dental and mandibular prosthetic implants. Today, the poly(methyl-methacrylate) (PMMA) resin is the most spread material, due to its ease of processing, low cost, aesthetic properties, low weight, biocompatibility, and biostability in the oral cavity. However, the porous surface (which favors the adhesion of microorganisms) and the weak mechanical properties (which lead to wear or fracture) are the major concerns. The inclusion of engineered nanomaterials in the acrylic matrix could improve the performances of PMMA. In this study, we added two different kind of nanomaterials, namely titanium dioxide nanoparticles (TiO_2_NPs) and halloysite clay nanotubes (HNTs) at two concentrations (1% and 3% *w*/*w*) in PMMA. Then, we assessed the effect of nanomaterials inclusion by the evaluation of specific physical parameters: Young’s modulus, roughness, and wettability. In addition, we investigated the potential beneficial effects regarding the *Candida albicans* (*C. albicans*) colonization reduction, the most common yeast responsible of several infections in oral cavity. Our experimental results showed an improvement of PMMA performance, following the addition of TiO_2_NPs and HNTs, in a dose dependent manner. In particular, the presence of TiO_2_NPs in the methacrylate matrix induced a greater increase in PMMA stiffness respect to HNTs addition. On the other hand, HNTs reduced the rate of *C. albicans* colonization more significantly than TiO_2_NPs. The results obtained are of great interest for the improvement of PMMA physico-chemical properties, in view of its possible application in clinical dentistry.

## 1. Introduction

The rehabilitation of masticatory function in edentulous patients, due to trauma, dental diseases or cancer, is one of the aspects of greatest interest in reconstructive medicine and dentistry. The need to improve the quality of oral prostheses and then, the quality of life of patients, has stimulated the research for biocompatible materials (non-toxic, non-irritating, and non-carcinogenic) able to ensure good structural and mechanical performance. In detail, an ideal biomaterial for dental and mandibular prostheses should be chemically stable, mechanically rigid, and non-deformable. In addition, prosthetic material should be resistant to impact and to mechanical stress arising from masticatory forces. Another point to consider is reletad to the aesthetic characheristics; ideally, the color of the material must resemble the color of the oral cavity as closely as possible. For that reason, it should be translucent. Finally, the material should be easily workable, repairable, and cleanable, as well as inexpensive.

Although numerous materials have been proposed over the past century [1,2,3], none of them meet the ideal characteristics described for basic oral prosthetic materials. Today, poly(methylmethacrylate) (PMMA), introduced by “Walter Wright” in 1937, is recognized to be the best with respect to other denture base materials for both complete and partial dentures [4]. PMMA is low cost, low weight, biocompatible, and biostable in oral cavity; in addition, it posses good aesthetic properties.

PMMA is an amorphous polymer formed by the polymerization of the monomer methylmethacrylate; its physical and mechanical properties can be affected by polymerization conditions and fabrication procedures. Several studies have shown that, for dental applications, PMMA requires hot polymerization [5]. In addition, it is also reported some critical issues regarding the processing method; the volatilization of the methylmethacrylate monomers and shrinkage processes that occur during polymerization, resulting in the formation of surface defects such as pores, cracks, and irregularities [6]. These defects, besides to induce deformation resistance of PMMA (i.e., reduction in elastic modulus), and consequently weak mechanical properties, including impact and flexural strengths [7]. At the same time, PMMA became the optimal surface for the growth and proliferation of microrganims [8,9]. Among several species, *Candida albicans* (*C. albicans*), an opportunistic yeast, causes the most numerous infections of the oral cavity [10,11]. Several studies reported the adhesion behavior of *C. albicans* on surface that significantly increases when the surface roughness is hight [12]; moreover, the superficial irregularities may protect the microorganisms from shear forces associated to the mechanical cleaning processes [13].

In the last decades, several studies have been carried out to improve the physico-chemical properties of polymeric materials, including PMMA; in particular, it has been demonstrated that the inclusion of engineered nanomaterials (nanoparticles, nanotubes, nanofibers) in the polymer matrix is capable to modify the material properties [14,15,16]. These alterations depend on the nanomaterials types, concentration, size, morphology, and surface charge [17,18].

For example, zirconium dioxide NPs (ZrO_2_NPs) were used in PMMA due to their biocompatibility and high strength, improving the mechanical properties of NPs in a dose-dependant manner [19]. However, this effect tended to be reduced when the concentration of NPs increased, provoking agglomeration phenomena that consequently resulted in the worsening of material [20]. Additionally, diamond nanoparticles (DNPs) were added in PMMA for their good thermal conductivity and hardness, but in same cases the aesthetic properties regarding the prosethesis color makes their application difficult for clinical trials [21,22,23].

Carbon nanotubes (CNTs) were recently applied in PMMA, due to their strong antimicrobial effects and the mechanical properties enhancement [24,25]. However, the breakage of the material due to wear can release CNTs that are know to be toxic, as demonstrated in previously works [26,27].

In this work, using atomic force microscopy (AFM), we evaluated the impact on elastic and surface properties of PMMA after addition by both synthesized titanium dioxide nanoparticles (TiO_2_NPs) and commercial halloysite clay nanotubes (HNTs) at two different concentrations (1% and 3% *w*/*w*).

These two nanomaterials were chosen for their unique physico-chemical properties; in particular, TiO_2_NPs have antibacterial and antimicrobial properties by oxygen radicles production able to destroy the cell wall of some kinds of bacteria inhibiting the biofilm formation [28,29]. Different results can be obtained tuning the NPs size and shape. In addition, the surface properties were responsible of the strong bonds with PMMA chemical groups enhancing the reinforcement effectiveness [30]. HNTs has a hollow/tubular structure constituted by two layers of aluminosilica [31]. CNTs are abundant and cheap natural minerals with biocompatible features [32].

Once analyzed the elastic properties, the change of surface roughness and surface contact angle values were estimated following the addition of TiO_2_NPs and HNTs in PMMA, in order to understand a possible contribution to reduce the *C. albicans* colonization rate and the cell morphology. The results obtained claim the possibility to customize PMMA with different kind of nanomaterials to enhance its efficiency in clinical dental applications.

## 2. Materials and Methods

### 2.1. Engineered Nanomaterials: Synthetized TiO_2_NPs and Commercial HNTs

TiO_2_NPs have been synthesized by the sol-gel route [33] with some modifications [34,35]. Titanium (IV) isopropoxide (TTIP, 99,9%, 377996, Merck, Darmstad, Germany), used as precursor, was dropped in a solution constituting of ethanol and milliQ water in a molar ratio of 5:1:1 under stirring in acidic conditions (pH 3). Then, NPs were heated for 5 h at 30 °C first, and then at 430 °C for 3 h in order to obtain a white nano powder.

The commercial HNTs (n. 685445, Merck, Darmstadt, Germany) were kindly donated by Prof. Yuri Lvov (Louisiana Tech University, Ruston, LA, USA).

### 2.2. Morphological and Compositional Characterization of TiO_2_NPs and HNTs

Scanning electron microscopy (SEM) and energy-dispersive X-ray spectroscopy (EDS) measurements were performed using a JEOL JSM-6480LV microscope (Jeol Inc, Peabody, MA, USA), with a magnification of up to 10,000×. The SEM images have been acquired at an accelerating voltage of 5 kV (in order to better distinguish the surface details), whereas EDX measurements need higher accelerating voltages in order to ionize the atoms in the sample and induce X-ray emission.

The samples were prepared dissolving 5 mg of TiO_2_NPs or HNTs in 10 mL of milliQ water. Then, after one sonication cycle (10 min, 59 MHz), the nanomaterials were deposited on monocrystalline silicon wafer by drop casting.

### 2.3. Fabrication of PMMA Based Samples

The PMMA based specimens were obtained by mixing, in a glass container, 5 g of Paladon 65 powder (Kultzer, Germany) and 3 mL of Paladon 65 liquid (Kultzer, Germany); they were mixed manually using a steel spatula until the mixture reached a semi-liquid consistency. After 3 min of rest, necessary for the resin to acquire a semi-plastic consistency, the mixture was poured into hard silicone moulds (80 shore hardness) and then transferred into a Pentatlon 205 machine (Effegi Brega, Sarmoto, Italy); here the polymerization of the samples was achieved in 30 min, setting the pressure at 5 atm and the temperature at 100 °C.

The cured PMMA specimens were treated then roughened, finished and polished. More specifically, the surfaces were polished using first a tungsten carbide multi-blade and then silicone rubbers of decreasing grain size (from 40–100 µm), mounted on a rotating instrument (SILFRADENT S.R.L, S. Sofia, Italy) (50,000 rpm). Subsequently, the surface was polished using a cotton-wool brush mounted on a laboratory cleaner (SILFRADENT S.R.L, S. Sofia Italy) with water and pumice powder, and finally rubbed with a dry cotton-wool brush polishing liquid for resins (Dentaurum, Bologna, Italy).

In the case of PMMA implemented with engineered nanomaterials, TiO_2_NPs (1% and 3% *w*/*w*) or HNTs (1% and 3% *w*/*w*) were added to Paladon 65 powder before adding the Paladon 65 liquid component.

### 2.4. Color Analysis

The PMMA discs were photographed, using a reflex digital camera (Canon EOS 100D, Tokyo, Japan) mounted on a homemade setup to ensure that the objective-sample distance was always the same and under the same lighting conditions.

For each samples, the values of the RGB color components were obtained using the ‘color histogram’ tool of the ImageJ 1.47v software (National Institutes of Health, Bethesda, MD, USA).

### 2.5. Wettability Characterization

Wettability measurements were performed according to the sessile drop method. The experimental setup used is equipped with high-speed b/w motion analysis (Redlake Imaging MotionScope (DEL imaging, Woodsville, NH, USA)), linear positioner (Holmark, Kalamassery, Kerala, India) with integrated tilting stage, led backlight diffuser (Edmund, Reno, NV, USA) with intensity control, and microliter droplets dispenser module (Hamilton, York, UK). The measurements, carried out at room temperature T = (20.5 ± 0.5) °C and relative humidity RH = (55 ± 5)%, were performed by depositing ≈ 3 µL of a drop of distilled water on the sample; making sure that the deposition of the drops occurred slowly, in order to prevent them from falling. Droplet deposition was recorded by frame grabber system from transient phase to steady-state configuration in which the static contact angle was measured.

For each of the sample (PMMA, PMMA + TiO_2_NPs 1%, PMMA + TiO_2_NPs 3%, PMMA + HNTs 1% and PMMA + HNTs 3%) 10 drops were used.

Recorded images were processed by using two different programs, Java plug-in of ImageJ 1.47v software (National Institutes of Health, Bethesda, MD, USA) and Ossila Contact Angle (Ossila, Sheffield, UK) [36]. This approach allowed to improve the static contact angle measurements, overcoming the limits related both to the asymmetry of droplets and to the sensitivity of image analysis techniques, including image contrast and left and right three-phase point identification, to reconstruct the contour based on the sharpness of grayscale pixels [37].

### 2.6. Atomic Force Microscopy (AFM) Analysis

High resolution AFM NTEGRA (NT-MDT Spectrum Instruments, Moskow, Russia) was employed to investigate morphology and roughness of samples. These measurements were performed using CSG01 probe (NT-MDT Spectrum Instruments, Moskow, Russia), These measurements were performed using CSG01 probe (NT-MDT Spectrum Instruments, Moskow, Russia). This tip have a tetrahedral shape, having a typical tip curvature radius smaller than 10 nm, and a tip cone angle less than 20°. The tip is mounted on a rectangular single crystal silicon cantilever with a thickness of 1.25 µm, a typical spring constant of about 0.03 N/m and resonant frequency is 15.06 kHz. The topographic acquisitions were taken in contact error mode, over areas of 50 µm × 50 µm with a resolution of 1024 points × 1024 points, with setpoint and gain parameters set to 1.1 nA and 0.34, respectively.

Further acquisitions were made on areas of 5 µm × 5 µm; these images were analyzed by Image Analysis P9 (NT-MDT Co.) software (NT-MDT Spectrum Instruments, Moskow, Russia) to accurately estimate roughness parameter, expressed as Rq and calculated as root mean square of height fluctuations respect to mean height value obtained by all data images. Prior to perform Rq quantification, each topographical image was treated with a second order plane fit and with a second order flattening in order to cancel every bow and tridimensionality effects.

In order to quantify the sample elasticity, force indentation curves were obtained using DPC20 probes (NT-MDT Spectrum Instruments, Moskow, Russia) that consists in high sensitivity V-shaped cantilever having tip cone angle less than 22°, curvature radius equal to 100 nm. The nominal resonant frequency and elastic spring constant indicated by the manufacturer amount to 420 kHz and 65 N/m, respectively. Therefore, prior to perform the force-spectroscopy measurements, the spring constant of DPC20 cantilevers was accurately estimated via the thermal noise method [38]. All measurements were performed in ambient conditions (room temperature of about 25 °C and relative humidity around 55%). The approach data portion of indentation curves were fitted using the Sneddon model and the Young’s Modulus (*E*) value was obtained as best fit parameter. For each sample, the *E* was calculated as mean value on 50 indentation curves.

### 2.7. C. albicans Colonization Analyses

*C. albicans* yeast working solution were obtained growing yeast cells in Sabouraud dextrose broth (SDB) (ThermoFisher, Waltham, MA, USA) for 18 h at 37 °C with shaking at 150 rpm. Prior to perform the yeast adhesion experiments, the *C. albicans* cells were harvested by centrifugation at 3000 rpm for 10 min; finally, cell density was adjusted to 1 × 10^7^ cells/mL in SDB.

1 mL of working solution were seeded on different PMMA-based samples (PMMA, PMMA+ TiO_2_NPs 1%, PMMA + TiO_2_NPs 3%, PMMA + HNTs 1%, PMMA + HNTs 3%), previously placed into 24-well tissue culture plate (Corning, St. Louis, MO, USA), in order to quantify the yeast colonization rate after incubation at 37 °C for 24 h and 48 h. At these time points, after gently washing with sterile PBS to remove non-adherent cells, adherent cells were fixed by immersion in methanol (50% *v*/*v*) for 2 min. After drying, samples were metallized with gold (approximately 10 nm thickness) before examination by scanning electron microscopy (JEOL USA, Inc, Peabody, MA, USA). The acquisitions obtained were analyzed with the open-source software ImageJ 1.47v software (National Institutes of Health, Bethesda, MD, USA), in order to quantify the percentage of colonized area.

In order to evaluate the morphological alterations of *C. albicans* yeast cells on different PMMA-based substrate, the atomic force microscopy surface characterizations were carried out in semicontact mode. The measurements were carried out using a single crystal silicon-antimony doped NGS 01 probe (NT-MDT Spectrum Instruments, Moskow, Russia), having a typical curvature radius of 10 nm, nominal resonant frequency of 150 kHz, and nominal elastic spring constant equal to 5.1 N/m.

The acquisitions were achieved, for both time points, over scan area equal to 50 over areas of 15 µm × 15 µm with a resolution of 768 points × 768 points;

In addition, further acquisitions were made on areas of 5 µm × 5 µm with resolution of 512 points × 512 points, in order to better appreciate the morphological changes in *C. albicans* yeast cells, after 24 h and 48 h. The parameters setpoint, gain and scan set parameters used were fixed to 4.5 nA, 0.3, and 0.5 Hz, respectively.

### 2.8. Statistical Analysis

Results were expressed as mean value and associated standard deviation. Differences between different mean values were considered statistically significant respect to control (PMMA substrate) performing a Student’s *t*-test with a *p*-value < 0.05 (<0.05 *, <0.01 ** and <0.005 ***).

## 3. Results and Discussion

Nowadays, PMMA is the common material used for oral prosthesis, despite there are some concerns regarding its suitability, due to the susceptibility to the fractures under the continuous action of chewing forces.

In this work, we evaluated the change in PMMA properties following its implementation with two different types of engineered nanomaterial: TiO_2_NPs and HNTs.

**Figure 1:**
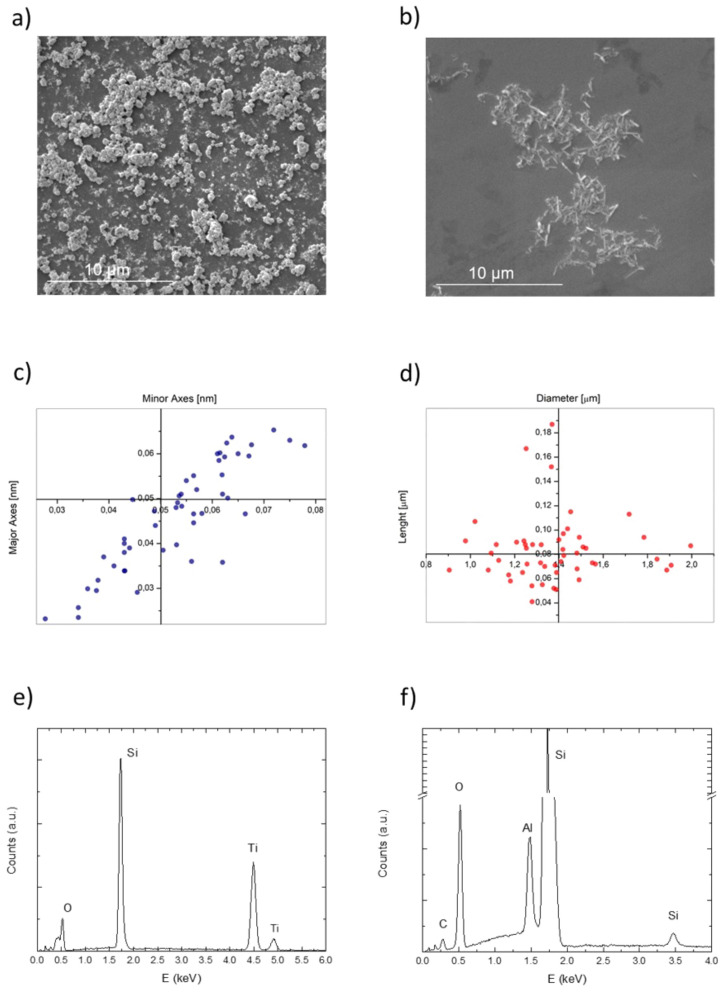
Representative SEM acquisitions of TiO_2_NPs (**a**) and HNTs (**b**) on monocrystalline silicon wafer at the accelerating voltage of 5 kV. Size distribution of TiO_2_NPs (**c**), in terms of major and minor axes length, and HNTs size distribution (**d**), in terms of diameter and lenght dimensions. EDS characterization (accelerating voltage 20 kV) of TiO_2_NPs (**e**) and HNTs (**f**).

We first characterized the nanomaterials using SEM to acquire information about their morphology (Figure 1).

TiO_2_NPs exhibited an irregular quasi-spherical shape (Figure 1a) with an average diameter of (50 ± 1) nm (Figure 1c); HNTs showed the standard nanotubes morphology (Figure 1b) with an average dimensions of (1.4 ± 0.2) µm in length and (0.08 ± 0.03) µm in height (Figure 1d), confirming the size value indicated by the manufacturer. Size statistical analysis of the NPs mean size and size distribution were performed by using the fitting analysis available on OriginPro software (OriginLab version 8, OriginLab Corporation, Northampton, MA, USA).

As showed in Figure 1c,d, the analyses corroborated the size distribution of the nanomaterials observed in SEM images, for TiO_2_NPs and HNTs respectively.

The EDS microanalysis conducted on TiO_2_NPs displayed the presence of titanium (Ti) and oxygen (O) in TiO_2_NPs and the absence of other chemical species (Figure 1e); the silicon (Si) peak was referred to the substrate (silicon wafer). Similarly, the EDS spectrum acquired for the HNTs samples showed peaks related to aluminium (Al), silicon and oxygen atoms (Figure 1f), in accordance with nanotubes composition. The peaks assigned to silicon in Figure 1f are due to the concurrent contributions of the nanotubes and the substrates.

**Figure 2:**
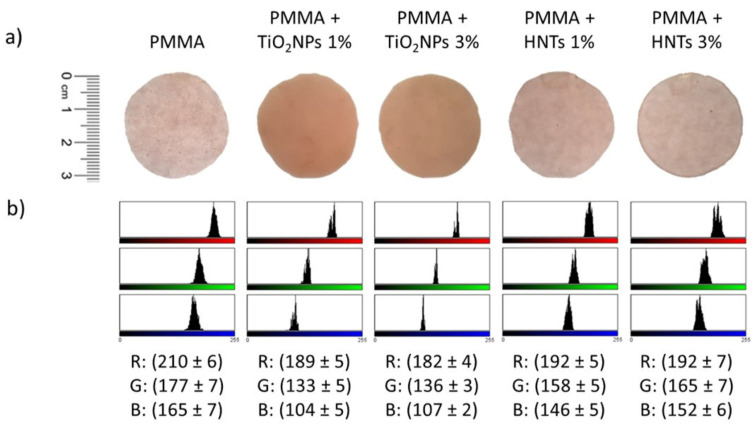
PMMA-based material samples images acquired with camera (**a**) and the corresponding RGB color analysis (**b**).

After characterization by SEM analysis, the two types of nanomaterials were added to the methyl-methacrylate resin at two different concentrations during sample preparation: 1% *w*/*w* and 3% *w*/*w*. After PMMA polymerisation, the nanomaterials were trapped in the methyl-methacrylate matrix. The first visible effect of their presence was the change in the color of the material (Figure 2).

The addition of HNTs did not alter the color of the PMMA, which remained pink and translucent like control, whereas the addition of TiO_2_NPs made the resin opaque (Figure 2a). These evidences were confirmed by RGB analysis, from which was evident how the value of red, green and blu channels after TiO_2_NPs addition (1% and 3%) changed respect to undoped PMMA (Figure 2b). Contrary, the HNTs did not produce significative changes in the three RGB channels (Figure 2b). The color change is a key aspect for aesthetic purposes because PMMA-based materials are destinated to be used in clinical practice. Subsequently, the impact of the nanomaterials addition in the methacrylate resin was evaluated in terms of elasticity and roughness using AFM.

**Figure 3:**
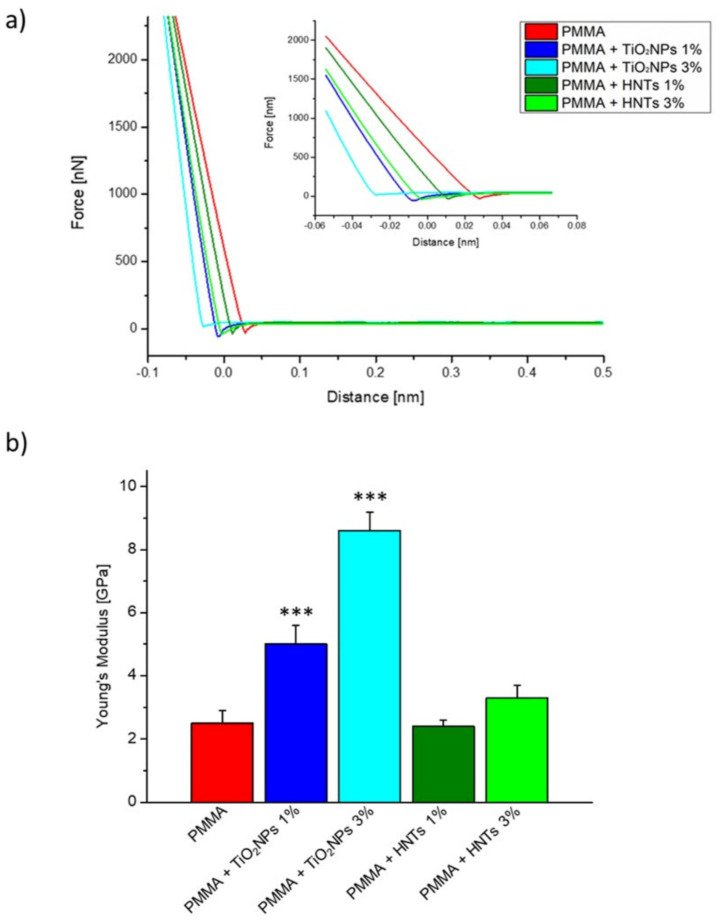
(**a**) Representative force-distance curves (only approach data portion) recorded on different PMMA-based materials. In the histogram (**b**) the mean value and its standard deviation of Young’s Modulus, evaluated for control (PMMA), PMMA + TiO_2_NPs 1%, PMMA + TiO_2_NPs 3%, PMMA+ HNTs 1%, and PMMA+ HNTs 3% were represented. Reported results were considered statistically significant respect to control (PMMA) for *p*-value < 0.005 ***.

The results obtained by Young’s modulus estimation (Figure 3) clearly demonstrated that the inclusion of HNTs or TiO_2_NPs triggered a stiffness alteration in the PMMA matrix, as suggested by the different slope of the linear approach portion in the force–distance curves (Figure 3a).

In details, the Young’s modulus estimated for the samples PMMA, PMMA + TiO_2_NPs 1%, PMMA + TiO_2_NPs 3%, PMMA + HNTs 1%, PMMA + HNTs 3% showed how the presence of nanomaterials in the resin increased the PMMA stiffness (Figure 3b), which was equal to (2.5 ± 0.4) GPa in the control sample, increasing considerably in the samples in which TiO_2_NPs was added, becoming equal to (5.0 ± 0.6) GPa in PMMA+ TiO_2_NPs 1% and (8.6 ± 0.6) GPa in PMMA+ TiO_2_NPs 3%.

The Young’s Modulus value in the PMMA + HNTs 1% sample did not change respect to the control, remaining equal to (2.4 ± 0.2) GPa, while a slight increase was recorded for PMMA + HNTs 3%, becoming equal to (3.3 ± 0.4) GPa.

The explanation of the best performance of TiO_2_ NPs in PMMA matrix could be due by a chemical and physical interactions with the carboxylic groups (-COOR) of PMMA in different manner. For example, the binding can occur by a bidentate coordination between Ti^4+^ cation and the two oxygen atoms of -COOR. Alternatively, the bond can take place between the hydroxyl group (-OH), exposed on TiO_2_NPs surface and carbonyl group (-C=O) of PMMA [39]. Moreover, the addition of TiO_2_NPs is particularly suitable to fill the interpolymeric space [40].

The HNTs filled in the matrix, and the potential agglomeration could decrease their degree of anisotropy due to the increase to the weak van der Walls interactions with the PMMA matrix [41].

The best values regarding the mechanical rigidity were recorded when TiO_2_NPs were added in PMMA (Figure 3b). In the case of HNTs, the increase of Young’s modulus value become significant using the concentration of 3% *w*/*w*. Contrary, the effect on the mechanical improvement of PMMA implemented with TiO_2_NPs was considerable even at the lowest concentration tested in this work (1% *w*/*w*).

**Figure 4:**
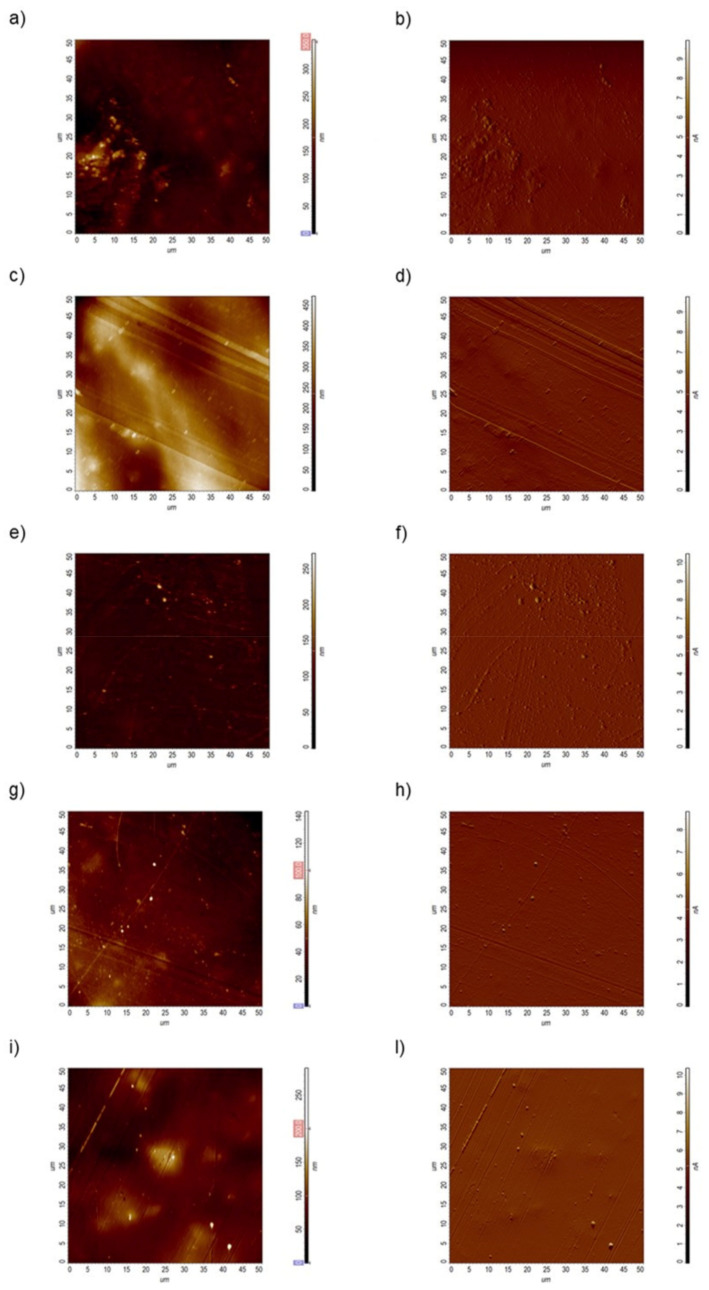
Representative topographical acquisitions obtained by atomic force microscopy in the height and deflection channel, obtained for PMMA (**a**,**b**), PMMA + TiO_2_NPs 1% (**c**,**d**), PMMA + TiO_2_NPs 3% (**e**,**f**), PMMA+ HNTs 1% (**g**,**h**), PMMA+ HNTs 3% (**i**,**l**) substrates.

High-resolution atomic force scans performed in contact error mode, permit to simultaneously estimate the height local changes (heigh channel) and to appreciate more details about the features present on the sample surface (DFL channel). The topographic acquisitions revealed that the PMMA surface became smoother when the engineered nanomaterials were included in the matrix (Figure 4).

**Figure 5:**
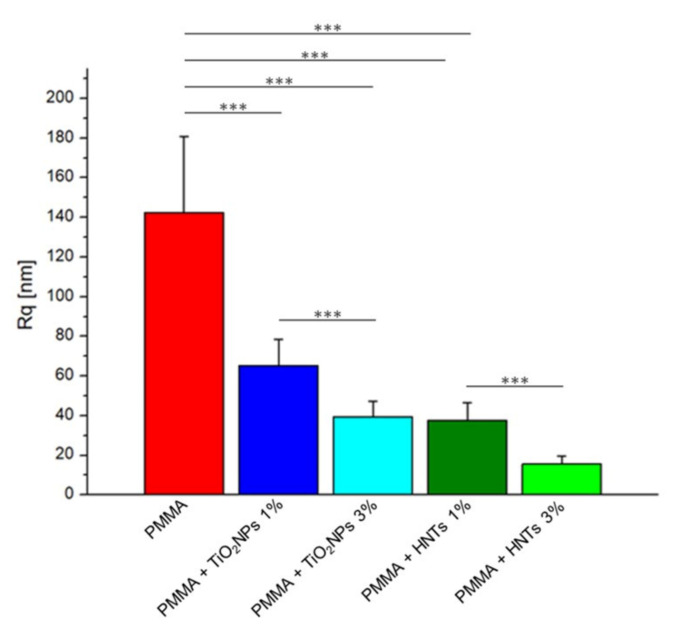
Roughness values (Rq) expressed as mean value and standard deviation on PMMA-based substrates. Obtained Rq value were considered statistically significant respect to control (PMMA) for *p*-value < 0.005 ***.

The analysis of the surface properties of the PMMA-based materials revealed a drastic reduction of the roughness parameter for both nanomaterials added to the methacrylate matrix, depending on their concentration (Figure 5).

As shown in the histogram, the roughness value of the PMMA sample, equal to (142 ± 38) nm was reduced by 54% when TiO_2_NPs 1% *w*/*w* was added; such value decreased up to 72% when the concentration of TiO_2_NPs was 3% *w*/*w*. Better results were recorded using PMMA + HNTs 1% and 3% samples, in which the Rq value decreased of 73% and 89% respect to the control sample.

**Figure 6:**
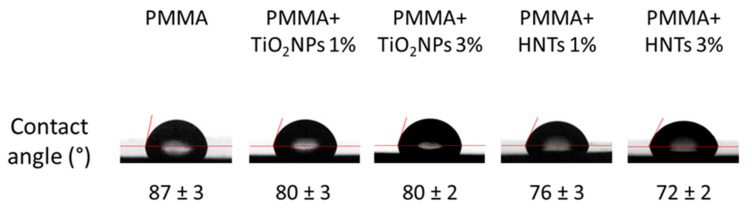
Water contact angle measurements. Representative images of water droplets on different PMMA-based materials and the correspondent contact angle values, obtained as mean on different 10 experiments ± sd.

In general, to test the wettability, the contact angle is measured. This parameter is described as the angle at the interface where water, air and solid converge. High contact-angle values of certain surfaces display the surface’s tendency to repel water; on the contrary, low values referred to the water tendency to spread on the surface [42]. By the contact angle measurements, we verified the wettable nature of PMMA after nanomaterials addition at 1% and 3% *w*/*w* (Figure 6).

More exactly, the value of the water contact angle recorded for PMMA sample (control) was equal to (87 ± 3)°, and it decreased when TiO_2_NPs or HNTs were added. More specifically, the values of this parameter become equal to (76 ± 3)° and (72 ± 5)° in PMMA+ HNTs 1% and 3% samples, respectively. Our results were in close agreement with the results reported by Wei and colleagues [43], although the nanotubes concentrations used were different from those used in our work.

The implementation of PMMA with TiO_2_NPs less significantly reduced the contact angle value, which was equal to 80° regardless of the concentration of NPs (1% and 3% *w*/*w*) added to the methacrylate matrix.

Thus, we found that the hydrophilicity increased in PMMA + HNTs than in PMMA + TiO_2_NPs samples, whereas the roughness value was smaller.

It has been largely demonstrated how the Rq and wettability parameters affect both the material biocompatibility and the possible pathogen or yeast colonization.

As reported in clinical literature, most oral infections developed by patients wearing dental or mandibular prostheses are due to the uncontrolled proliferation of the yeast *C. albicans* [44,45]. This yeast is able to reduce the lifetime of the prosthetic implant and in addition to this, it strongly worsens the health of the oral cavity negatively impacting on the patient’s quality of life.

**Figure 7:**
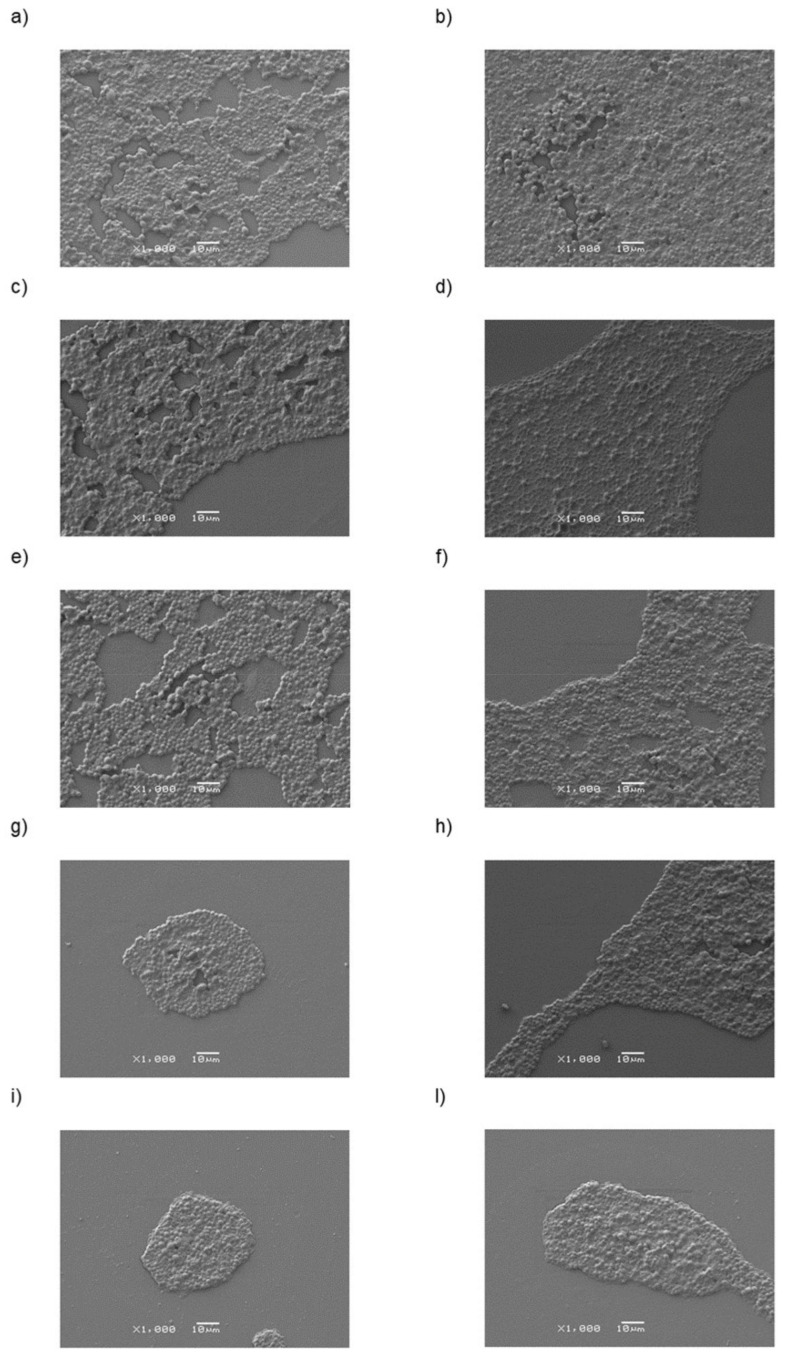
Representative SEM images at 1000× magnifications of *C. albicans* presence on different PMMA-based substrates: PMMA (**a**,**b**), PMMA + TiO_2_NPs 1% (**c**,**d**), PMMA + TiO_2_NPs 3% (**e**,**f**), PMMA+ HNTs 1% (**g**,**h**), PMMA+ HNTs 3% (**i**,**l**) substrates at different time points 24 h (**a**,**c**,**e**,**g**,**i**) and 48 h (**b**,**d**,**f**,**h**,**l**).

SEM acquisitions (Figure 7) suggested that the rate of colonization by *C. albicans* yeast decreased on PMMA substrates implemented with TiO_2_NPs and this reduction become pronounced in PMMA + HNTs samples.

**Figure 8:**
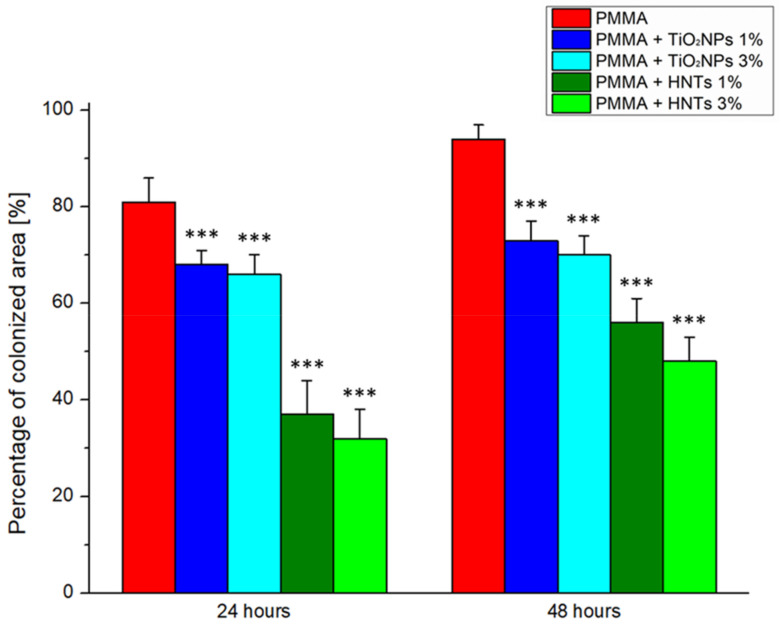
Histograms reported the Colonization assay measurements of *C. albicans* on different PMMA-based substrates. The colonized area was expressed as a percentage rate of the *C. albicans* covered area respect to entire acquired surface at two time points (24 h and 48 h). Reported results were calculated as average ± SD on three different area on each sample, and the values were considered statistically significant respect to control (PMMA at correspondent time point) for *p*-value < 0.005 ***.

Following the SEM images analysis byusing ImageJ software, the percentage of colonized area after 24 h on the PMMA + TiO_2_NPs 1% and 3% samples was reduced by 16% and 19%, respectively, compared to the control sample. After 48 h, the percentage of colonisation respect to undoped PMMA further decreased to 22% and 26% on the PMMA + TiO_2_NPs 1% and 3% substrates, respectively (Figure 8).

The addition of HNTs to methacrylate resin inhibited the Candida colonization in a stronger manner than the undoped PMMA.

**Figure 9:**
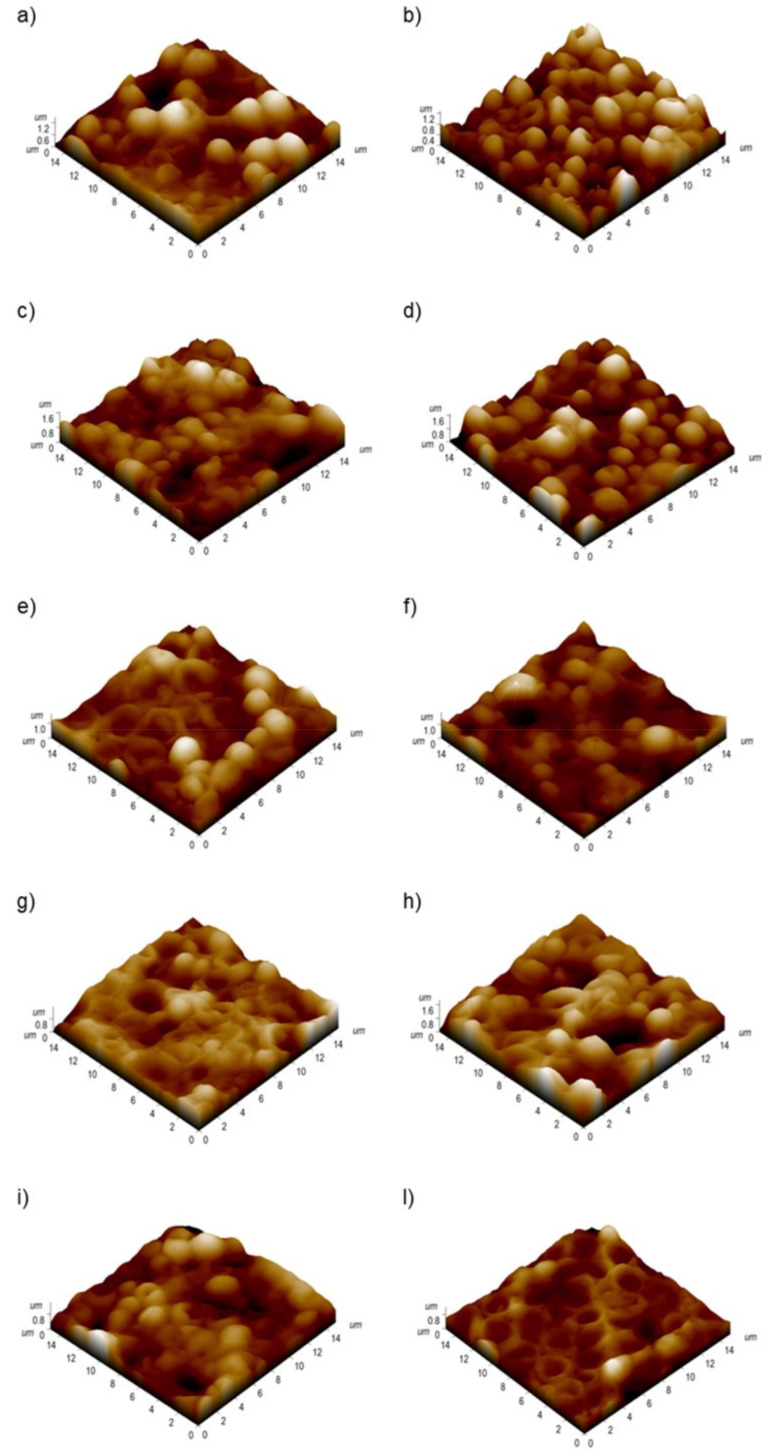
Three dimensional AFM topographical acquisitions, performed on PMMA (**a**,**b**), PMMA + TiO_2_NPs 1% (**c**,**d**), PMMA + TiO_2_NPs 3% (**e**,**f**), PMMA+ HNTs 1% (**g**,**h**), PMMA+ HNTs 3% (**i**,**l**) substrates after 24 h (**a**,**c**,**e**,**g**,**i**) and 48 h (**b**,**d**,**f**,**h**,**l**) of *C. albicans* colonization.

After 24 h, the percentage of colonized area on the PMMA + HNTs 1% and 3% substrates was reduced by 54% and 60% compared to the control. At 48 h, this reduction become equal to 40% for PMMA + HNTs 1% and 49% for PMMA + HNTs 3%. These results, obtained on three different areas on each sample, were corroborated by morphological observations of *C. albicans* cells, obtained by AFM acquisitions (Figure 9).

The implementation of PMMA with TiO_2_NPs (1% and 3%) did not show any significant alteration, indeed the morphology of fungus appeared similar to untreated sample, in both time points. Contrary, the addition of HNTs both at 1% and 3% *w*/*w* produced visible alterations of cells morphology: *C. albicans* appeared collapsed and deformed with irregular shape. These effects become more pronounced after 48 h (Figure 9).

In summary, we concluded that the inclusion of engineered nanomaterials can improve the performance of PMMA for its use in buccal prosthetic implants. The addition of TiO_2_NPs increased the mechanical stiffness of the methacrylate resin, providing an improved wear and fracture resistance, and decreased the PMMA roughness. However, this additive dulls the methacrylate resin, making it less aesthetically pleasing. In contrast, the PMMA matrices implemented with HNTs preserved the translucency of the methacrylate resin but weakly improves its mechanical properties. The major advantage of this additivation is related to the strongly reduction in the *C. albicans* ability to adhere and proliferate, thank to the lowest surface roughness and more hydrophilic behavior [46,47]. In addition, the low toxic profile of these kind of NPs on living cells, make sure that they are the best choice to customize PMMA, compared to other types of nanomaterials [48,49,50,51].

## 4. Conclusions

In recent years, the scientific research has focused great attention to improve the performance of materials used for prosthetic applications. In particular, there are aneed to develop strategies to inhibit *C. albicans* adhesion and proliferation, which represent the main cause of deterioration in oral prosthetic implants.

Our work outline interesting perspectives in the reconstructive dentistry field, suggesting as the implementation of nanomaterials can be a useful strategy to improve PMMA properties. In the light of the reported results, we encourage the HNTs addition especially to prevent the *C. albicans* infections.

In the future, clinical trials could be performed in order to evaluate the impact of natural oral biofilm and pH condition on HNTs-doped PMMA properties.

## Data Availability

Not applicable.
